# Listening to Music during Warming-Up Counteracts the Negative Effects of Ramadan Observance on Short-Term Maximal Performance

**DOI:** 10.1371/journal.pone.0136400

**Published:** 2015-08-24

**Authors:** Asma Aloui, Walid Briki, Hana Baklouti, Hamdi Chtourou, Tarak Driss, Anis Chaouachi, Karim Chamari, Nizar Souissi

**Affiliations:** 1 Research Laboratory “Sport Performance Optimization”, National Center of Medicine and Sciences in Sport, Tunis, Tunisia; 2 High Institute of Sport and Physical Education of Gafsa, University of Gafsa, Gafsa, Tunisia; 3 Qatar University, College of Arts and Sciences, Sport Science Program, Doha, Qatar; 4 High Institute of Sport and Physical Education of Sfax, University of Sfax, Sfax, Tunisia; 5 Laboratoire CeRSM (EA 2931), Equipe de Physiologie, Biomécanique et Imagerie du Mouvement, UFR STAPS, Université Paris Ouest Nanterre La Défense, Nanterre, France; 6 Athlete Health and Performance Research Center, ASPETAR, Qatar Orthopedic and Sports Medicine Hospital, Qatar; University of L'Aquila, ITALY

## Abstract

**Aim:**

The aim of the present study was to examine whether listening to music during warming-up might influence short-term maximal performance (STMP), cognitive anxiety, self-confidence, and enjoyment during Ramadan, and whether these affects might predict STMP.

**Methods:**

Nine male physical education students (age: 21 ± 1.1 years; height: 1.8 ± 0.04 m; body mass: 83 ± 5 kg) volunteered to participate in the present study. A within-subjects design consisted of four experimental sessions: Two sessions occurred one week before Ramadan and two others took place during Ramadan. They were scheduled at 5 p.m. and were conducted as follows: After a 10-minute warm-up either with or without listening to music, each participant performed a 5-m multiple shuttle run test, after which he was asked to answer items intended to assess his affective state during the experimental task.

**Results:**

Our findings revealed that STMP was lower during Ramadan than before Ramadan in the no-music condition. Additionally, it was found that STMP was higher in the music condition than in the no-music condition during Ramadan, and that STMP measured before Ramadan did not differ from that measured during Ramadan in the music condition. Regarding affects, the findings revealed that enjoyment was lower during Ramadan than before Ramadan in the music condition, and that cognitive anxiety was lower in the music condition than in the no-music condition before Ramadan. Self-confidence was not influenced by the experimental conditions.

**Conclusion:**

This study showed that listening to music during warming-up not only would be beneficial for STMP in Ramadan fasters, but also would counteract the negative effects of Ramadan observance on STMP.

## Introduction

Ramadan is one of the most important celebrated religious traditions in the world during which healthy Muslims are required to abstain from eating, drinking, and some other behaviors during the daylight hours. This fast duration, depending on the geographical location and the season, may be as long as 18 hours per day in the summer of temperate regions. During latter years, Ramadan arrived in summer while many sport events coincided with the holy month (e.g., London 2012 Olympics, Brazil 2014 FIFA World Cup). As a result, Muslim athletes have to deal with a strong dehydration―induced by daytime fasting and a higher sweat-loss rate caused by hot climatic conditions―that may alter their sport performance [1].

Previous studies have demonstrated that short-term maximal performance (STMP) was lower during Ramadan than out-of-Ramadan control period when test sessions were conducted in the afternoon [2–6]. Using repeated sprint tests (*5 × 6-s maximal sprints* [2, 4, 5]; 6 × *15-m maximal sprints* [7]), authors found that peak power was lower during Ramadan than before Ramadan, suggesting that Ramadan may have negative effects on STMP. Although sport events usually take place after sunset during Ramadan in Muslim-majority countries, most of international sport events are rather dependent on television schedules, and cannot take into account Ramadan constraints. Therefore, to cope with Ramadan fasting, Muslim athletes have to find strategies. Because many studies revealed that listening to music might facilitate sport performance [8, 9], the present study focused on the link between music, affects, and STMP during Ramadan.

Most studies have used music during the ongoing performance and showed that listening to music might increase motivation [8, 10, 11], arousal [12], motor coordination [13], and improve maximal [8] and sub-maximal performance [9, 14]. It was also found that music might enhance positive affects (e.g., vigor, happiness) and reduce negative affects (e.g., anger, anxiety, depression) as well as perceptions of effort, fatigue, or pain [12, 15–17]. However, a few studies focused on the impact of listening to music during warming-up on subsequent exercise performance and showed beneficial effects on STMP [18, 19]. Additionally, given that athletes are not allowed to listen to music during competitions due to the sport-specific rules, examining the effects of listening to music during warming-up on sport performance would be helpful for fasters during Ramadan. As a result, the present study sought to examine the effects of listening to music during warming-up on STMP and affects.

Cognitive anxiety (i.e., worry or negative concerns about performance), self-confidence (i.e., degree of certainty about one’s ability to succeed a task), and enjoyment (i.e., enthusiasm for achieving a task) are important performance-related affects in sport [20–22]. Sport psychologists brought many evidences concerning the positive relationship between self-confidence and performance, and the negative relationship between cognitive anxiety and performance [22]. Likewise, positive links between enjoyment and performance were found [23–25]. However, there was no empirical research on how music can influence cognitive anxiety, self-confidence, and enjoyment. Therefore, the present study aimed to provide insight into the effect of listening to music on these performance-related affects and STMP during Ramadan.

### Study Overview

The literature reveals that the link between music and STMP in the context of Ramadan intermittent fasting, as well as its affective and motivational underlying mechanisms, still needs to be explored. Therefore, the purpose of the present study was to examine whether music might impact STMP (during a 5-m shuttle run test), cognitive anxiety, self-confidence, and enjoyment during Ramadan, and whether these affects might predict STMP. Three hypotheses were formulated. First, given that music was found to be positively related to positive affects and performance [8], but negatively related to negative affects [17, 26], it was predicted that self-confidence, enjoyment, and STMP would be higher in the music condition than in the no-music condition, while cognitive anxiety would display a reverse pattern. Second, given that Ramadan was found to decrease performance [2–7], and given the relationships between affects and performance [20, 22], it was predicted that self-confidence, enjoyment, and STMP would be lower during Ramadan than before Ramadan, while cognitive anxiety would be higher in the former condition than in the latter condition. Third, and consistent with our expected findings, it was predicted that self-confidence and enjoyment would be positively related to STMP, whereas cognitive anxiety would be negatively related to STMP.

## Materials and Methods

### Participants

Nine male physical education students (age: 21 ± 1.1 years; height: 1.8 ± 0.04 m; body mass: 83 ± 5 kg) volunteered to contribute to the present study. They were recruited because they declared that they were willing to fast the whole month of Ramadan, they were untrained, and that they had no current musculoskeletal injury.

### Experimental design

The Clinical Research Ethics Committee of the National Center of Medicine and Sciences in Sport of Tunis approved the experimental design of the present study. The study used a within-subjects design consisting of four experimental sessions: Two sessions occurred one week before Ramadan and two others at the mid-point of Ramadan. Before and during Ramadan, the two sessions were scheduled at 05:00 p.m. and were separated by a recovery period of 48 hours. In each period, the experimental task was preceded by either a warm-up period with listening to music (i.e., music condition) or a warm-up period without listening to music (i.e., no-music condition); technically, the use of a randomizer allowed us to compute our randomizations. When a participant was exposed to one music condition during the first session, he was exposed to the other one during the second session.

The month of Ramadan during which the present study was carried out was that of the year 2013. The elapsed time from dawn to sunset was from 03:28 a.m. to 07:33 p.m. at the beginning of Ramadan, and from 03:58 a.m. to 07:14 p.m. at the end of Ramadan. During that Ramadan, the participants took their last meal approximately at 02:00 a.m. and they fasted from that time until sunset.

### Procedure

Before the beginning of the experiment, the participants were familiarized with the experimental protocol, but received no information about the purposes of the present study until they fully completed the experiment. They were assured that their responses would remain confidential and signed an informed consent form. During each experimental session, and according to his assigned condition, each participant carried out a 10-minute warm-up either with or without listening to music, and performed a 5-m multiple shuttle run test [27]. Right after finishing his efforts, he was asked to answer items intended to assess his affective state during the experimental task (see Measures section below). When an experimental session was terminated, the participants were thanked; and when the entire experiment was terminated, they were fully debriefed and thanked for their participation.

#### Music conditions

In the music condition, the music played for each participant was self-selected. Indeed, the participants were asked to download their own music and to specifically select music on which they feel more inclined to do high intensity exercise. This procedure was based on studies that have shown that allowing participants to choose their own music had greater benefits on their performance than assigning pre-selected music to participants [28, 29]. Given the high intensity of the experimental task, all participants have chosen high tempo music (from 120 to 140 bpm) [8]. Music was played from an mp3 player through personal headphones, and was switched off after the 10-minute warm-up period. In the no-music condition, headphones were worn but no music was played.

#### Experimental task

The experimental task consisted in a 5-m multiple shuttle run test [27]. Six beacons were placed 5 m apart in a straight line to cover a total distance of 25 m. Each participant began the test in line with the first beacon (A); then, upon an auditory signal, he sprinted 5 m to a second beacon (B), touched the ground adjacent to that beacon with his hand, and returned back to (A), touching down on the ground adjacent to the beacon with his hand again. Then, he sprinted 10 m to a third beacon (C) and back to (A), etc., until 30 s of exercise had been completed. He was then allowed 35 s of recovery, during which he walked back to beacon (A). The 30-s run was performed six times [27]. The participants were instructed to perform maximally throughout the test.

### Measures

#### Physical Performance

The distance covered by each participant was recorded to the nearest 2.5 m during each 30-s run. STMP was assessed through calculating total distance, corresponding to the distance covered over the 6 × 30-s shuttles (see the data in S1 Text).

#### Affects

To measure enjoyment, the 7-item enjoyment subscale of the Enjoyment Inventory [30] was used. The items (e.g., “While I was doing this activity, I was thinking about how much I enjoyed it”) were rated on a 7-point Likert scale, from “*not at all true*” (1) to “*very true*” (7), with a mid-point “*somewhat true*” (4). Single items of cognitive anxiety and self-confidence, from the Mental Readiness Form-Likert (MRF-L, [31]), were also used. Specifically, the participants were asked to rate their thoughts on 11-point scales, from “*calm*” (1) to “*worried*” (11) for cognitive anxiety, and from “*confident*” (1) to “*scared*” (11) for self-confidence. We used single items to facilitate the assessment of feelings. Studies have ever used the scales from the MRF-L in sport settings [20, 21]. The data of enjoyment, cognitive anxiety, and self-confidence are available in S1 Text.

### Analyses

To examine our hypotheses regarding the effects of Music and Ramadan period on enjoyment, cognitive anxiety, self-confidence, and STMP, factorial analyses were conducted. However, before conducting such analyses, the normality of distributions was tested with Shapiro-Wilk’s test. When Shapiro-Wilk’s test was not significant (*p* > 0.05), meaning that the normality assumption was not violated, a two-way ANOVA with repeated-measures on both factors (Ramadan period [Before *vs*. During] × Music [With *vs*. Without]) was conducted on the variable of interest, and follow-up post-hoc comparisons (LSD Fisher tests) were conducted when appropriate. However, when Shapiro-Wilk’s test was significant (*p* < 0.05), a non-parametric test was conducted. Specifically, a Friedman test was used to examine the effect of experimental conditions. Follow-up pairwise comparisons were also conducted when appropriate. Preliminary findings revealed that the normality of data distribution was assumed for STMP only. Furthermore, to examine the relationships between affects and STMP, and given that the normality of data distribution was not assumed for affects, non-parametric correlation analyses were conducted with Spearman’s rho (ρ) test.

## Results

### Factorial analyses

#### Physical performance

**Fig 1:**
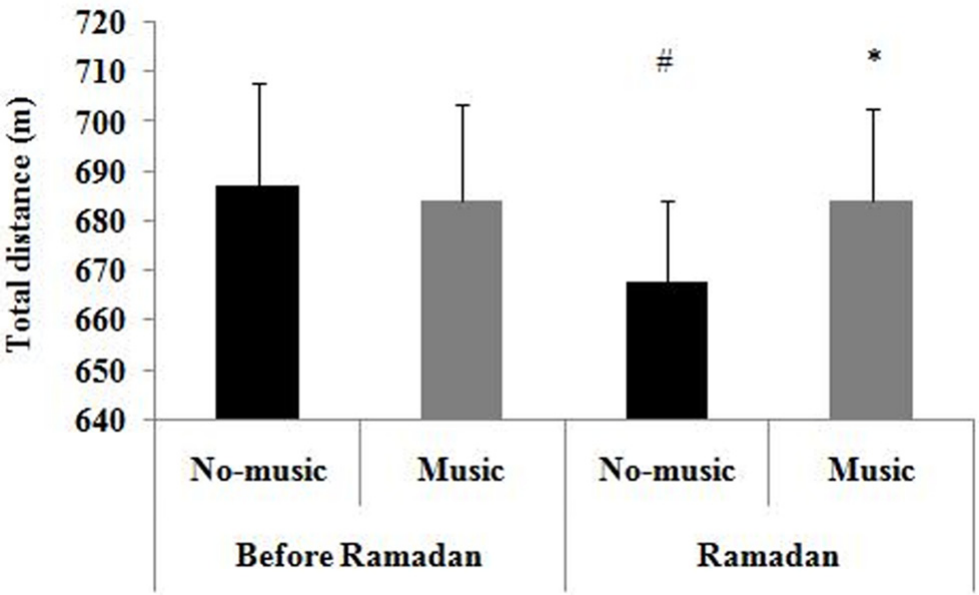
Means and standard deviations for total distance according to Music and Ramadan period conditions. #: significantly different compared to before Ramadan for the same music condition. *: significantly different compared to no-music condition during the same period.

A two-way ANOVA conducted on STMP revealed a significant Ramadan period × Music interaction, *F*(1,8) = 6.25, *p* = 0.04, η_p_^2^ = 0.44. Fisher LSD post-hoc tests revealed that: (a) in the no-music condition, STMP was higher before Ramadan than during Ramadan (*p* = 0.008) (Fig 1), (b) in the music condition, STMP measured before Ramadan did not differ from that measured during Ramadan (*p* = 0.99), and (c) during Ramadan, STMP was higher in the music condition than in the no-music condition (*p* = 0.02). Main effects of Music (*p* = 0.27) and Ramadan period (*p* = 0.09) were not significant (all *p*s > 0.05).

#### Affects

**Fig 2:**
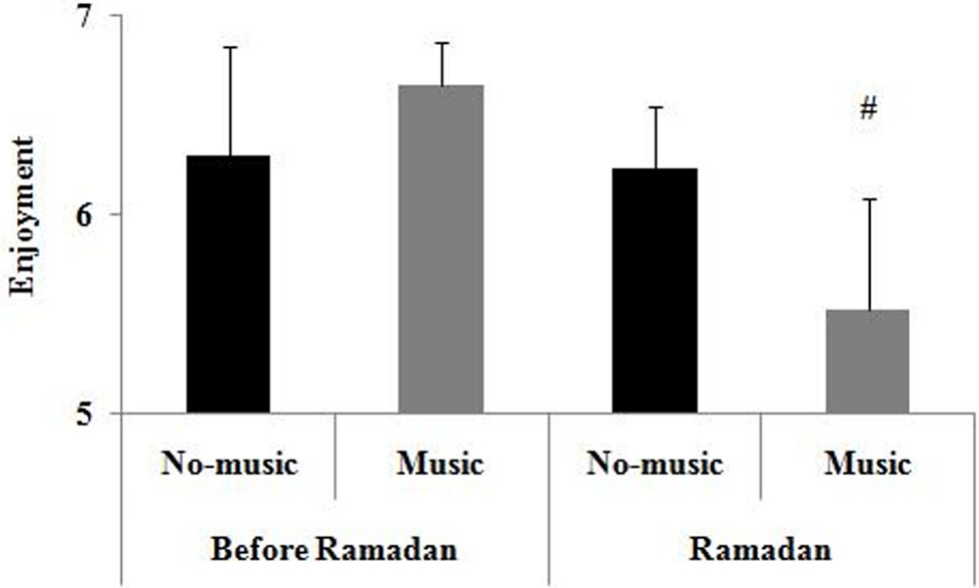
Means and standard deviations for enjoyment according to Music and Ramadan period conditions. On the y-axis, the number 1 represents the label “*not at all true*”, whereas the number 7 represents the label “*very true*”. #: significantly different compared to before Ramadan for the same music condition.

A Friedman test conducted on enjoyment revealed a significant effect of experimental conditions (test = 19.94, *p* < 0.001), and pairwise comparisons revealed that enjoyment was higher before Ramadan than during Ramadan in the music condition (*p* < 0.05) (Fig 2).

**Fig 3:**
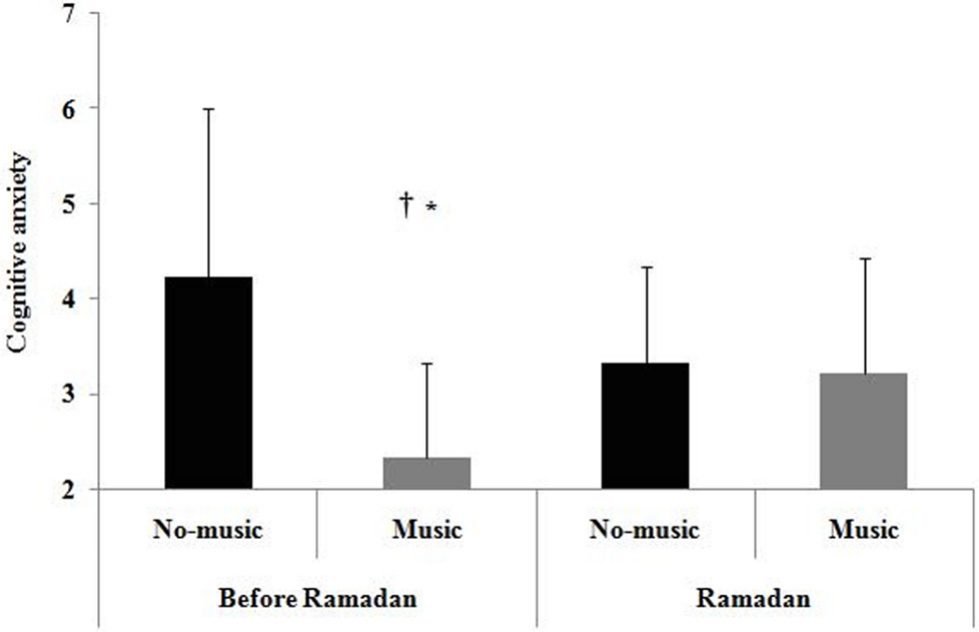
Means and standard deviations for cognitive anxiety according to Music and Ramadan period conditions. On the y-axis, the number 1 represents the label “*calm*”, whereas the number 11 represents the label “*worried*”. *: significantly different compared to no-music condition during the same period. †: significantly different compared to no-music condition of the other period.

A Friedman test conducted on cognitive anxiety revealed a significant effect of experimental conditions (test = 17.88, *p* < 0.001). Pairwise comparisons revealed that cognitive anxiety was (a) lower in the music condition than in the no-music condition before Ramadan (*p* < 0.05), and (b) lower in the music condition before Ramadan than in the no-music condition during Ramadan (*p* < 0.05) (Fig 3). Additionally, cognitive anxiety measured in the no-music condition did not differ from that measured in the music condition during Ramadan (Fig 3).

Regarding self-confidence, although a Friedman test revealed a significant effect of experimental conditions (test = 12.04, *p* = 0.007), subsequent pairwise comparisons revealed no significant differences between the conditions.

### Correlation analyses

In an attempt to provide evidence for our proposed motivational mechanism, correlation analyses examining relationships between affects and STMP were conducted. A global analysis conducted on the data set (*n* = 36) revealed no significant correlations between affects and STMP (enjoyment-STMP: ρ = 0.18; anxiety-STMP: ρ = 0.09; self-confidence-STMP: ρ = -0.03) (*p*s > 0.05).

## Discussion

The aim of the present study was to examine whether listening to music during warming-up might influence physical performance and affects in a shuttle run test carried out before and during Ramadan. Three hypotheses were formulated. First, it was expected that self-confidence, enjoyment, and STMP would be higher in the music condition than in the no-music condition, while cognitive anxiety would be lower in the music condition than in the no-music condition. Second, it was expected that self-confidence, enjoyment, and STMP would be lower during Ramadan than before Ramadan, while cognitive anxiety would be higher during the holy month. Third, it was expected that enjoyment and self-confidence would positively predict STMP, while cognitive anxiety would negatively predict STMP.

With regard to the first hypothesis, our findings revealed that cognitive anxiety was lower in the music condition than in the no-music condition before Ramadan, supporting previous studies that have evidenced a beneficial influence of music on affects and mood [8, 16, 17]. However, before Ramadan, enjoyment, self-confidence, and STMP did not change as a function of the music conditions, running counter to previous studies showing that listening to music might enhance STMP [8]. The absence of a music effect on these variables could be due to the fact that the participants listened to music while warming-up and not while performing the task. In accordance with this suggestion, authors [32] have recently shown that the greater the time interval after the use of a mental strategy, the lower the impact of this strategy on STMP.

Regarding the second hypothesis, the findings revealed that STMP was lower during Ramadan than before Ramadan in the no-music condition. This finding is consistent with those of previous studies showing that STMP was reduced during Ramadan [2, 4, 5, 7]. More specifically, these studies suggested that Ramadan observance would deplete physiological resources, thereby leading to a decrease in performance. Our findings also showed that enjoyment was lower during Ramadan than before this month in the music condition, suggesting that Ramadan would reduce the beneficial impact of listening to music on affects. Moreover, our findings revealed that: (a) during Ramadan, STMP was higher in the music condition than in the no-music condition, and (b) in the music condition, STMP measured before Ramadan did not differ from that measured during Ramadan. Taken together, these findings suggest that listening to music during warming-up would counteract the negative effects of Ramadan observance on STMP in fasters.

Correlation analyses revealed no significant relationships between affects and STMP, thus did not enable us to provide evidence for motivational mechanism (even if enjoyment was positively and slightly related to STMP).

This study is not without limitations. Indeed, this study was conducted on a relatively small number of participants, and this could explain the absence of some significant results. Consequently, further studies should use larger samples to test the robustness of findings with greater power, and to examine whether affects would mediate the relationship between listening to music and STMP during Ramadan. Moreover, caution should be taken when generalizing our conclusions since the sample used in this study included untrained participants only. In this context, it has been shown that untrained participants were more inclined to benefit from positive effects of listening to music than trained participants (e.g., [33]). Thus, further studies should test the moderating effect of the training status of participants using music in the context of Ramadan observance. Finally, further studies should take into account additional measures, such as physiological parameters (e.g., heart rate, $\dot{\text{V}}{\text{O}}_{2\text{peak}}$).

## Conclusion

This study represents a first attempt to examine the effects of listening to music during warming-up on STMP during Ramadan. The findings showed that music might be beneficial for STMP during Ramadan, and might counteract the negative effects of Ramadan observance on STMP. Thus, from a practical standpoint, listening to music during warming-up seems to be an effective strategy to counteract the negative effects of Ramadan observance on sport performance.

## Supporting Information

S1 Text Data of the study. (XLS)
